# Effectiveness of high-intensity interval training on glycemic control and cardiorespiratory fitness in patients with type 2 diabetes: a systematic review and meta-analysis

**DOI:** 10.1007/s40520-018-1012-z

**Published:** 2018-07-30

**Authors:** Jing-xin Liu, Lin Zhu, Pei-jun Li, Ning Li, Yan-bing Xu

**Affiliations:** 1 School of Kinesiology, Shanghai University of Sport, Shanghai, 200438 China; 2 Guangzhou Sport University, Guangzhou, 510500 China; 3 Department of Child Health Care and Rehabilitation, Lanzhou University Second Hospital, Lanzhou, 730030 China

**Keywords:** High-intensity interval training, Glycemic control, Cardiorespiratory fitness, Type 2 diabetes

## Abstract

**Aims:**

The aim of this systematic review and meta-analysis was to quantify the effect of high-intensity interval training (HIIT) on glycemic control and cardiorespiratory fitness compared with moderate-intensity training (MICT) and no training at all in patients with type 2 diabetes (T2D).

**Methods:**

Relevant articles were sourced from PubMed, Embase, the Web of Science, EBSCO, and the Cochrane Library. Randomized-controlled trials were included based upon the following criteria: participants were clinically diagnosed with T2D, outcomes that included glycemic control (e.g., hemoglobin A1c); body composition (e.g., body weight); cardiorespiratory fitness (e.g., VO_2peak_) are measured at baseline and post-intervention and compared with either a MICT or control group.

**Results:**

Thirteen trials involving 345 patients were finally identified. HIIT elicited a significant reduction in BMI, body fat, HbA1c, fasting insulin, and VO_2peak_ in patients with type 2 diabetes. Regarding changes in the body composition of patients, HIIT showed a great improvement in body weight (mean difference: − 1.22 kg, 95% confidence interval [CI] − 2.23 to − 0.18, *P* = 0.02) and body mass index (mean difference: − 0.40 kg/m^2^, 95% CI − 0.78 to − 0.02, *P* = 0.04) than MICT did. Similar results were also found with respect to HbA1c (mean difference: − 0.37, 95% CI − 0.55 to − 0.19, *P* < 0.0001); relative VO_2peak_ (mean difference: 3.37 ml/kg/min, 95% CI 1.88 to 4.87, *P* < 0.0001); absolute VO_2peak_ (mean difference: 0.37 L/min, 95% CI 0.28 to 0.45, *P* < 0.00001).

**Conclusions:**

HIIT may induce more positive effects in cardiopulmonary fitness than MICT in T2D patients.

## Introduction

Type 2 diabetes (T2D) is a metabolic disease characterized by hyperglycemia resulting from a resistance to insulin or a relative insulin insufficiency that can induce cardiovascular disease and lead to cardiovascular deterioration. According to epidemiological survey results, more than 422 million people worldwide were living with diabetes in 2014 [1], with a predicted prevalence of 552 million by 2030 [2]. Because of the growing economic and social burdens associated with T2D treatment, effective and accessible lifestyle interventions for people with T2D have never been more important. Exercise intervention is recognized as an integral concept for lifestyle intervention in T2D patients [3, 4], and it has been recommended by both the American Diabetes Association and the American College of Sports Medicine that patients should perform at least 150 min/week of moderate-to-vigorous aerobic exercise [5, 6]. Abundant evidence from randomized-controlled trials (RCTs) shows the benefits of aerobic exercise in glycemic control; for example, it reduces fasting glucose and improves insulin sensitivity, both of which help to alleviate the development of diabetes complications and mortality [7–10]. Furthermore, a recent meta-analysis demonstrated that aerobic exercise training is associated with a decrease in HbA1c, insulin resistance, and fasting glucose, and suggested that high-intensity aerobic exercise is superior to lower intensity exercise in improving cardiorespiratory fitness in T2D patients [11]. However, the majority of patients do not typically achieve the recommended level of physical activity, despite the fact that increases in physical activity level can improve glycemic control and cardiorespiratory fitness in T2D patients. In addition, a lack of time has been identified as one of the key barriers preventing patients from performing sufficient physical activity, which means that patients must participate in more time-efficient training programs to achieve optimized outcomes.

High-intensity interval training (HIIT), therefore, appears to be a feasible and time-efficient alternative exercise protocol to aerobic exercise: it involves alternating, repetitive short bouts of high-intensity exercise interspersed with less active or passive recovery periods. Numerous recent studies have shown HIIT to be superior in improving health benefits compared with lower intensity aerobic exercise in a variety of populations [12–14]. Støa et al. [15] found that people with T2D who performed a supervised HIIT program at an intensity of 85–95% of their maximal heart rate with 52% VO_2peak_ interval experienced a significant increase in VO_2peak_ and a reduction in hemoglobin A1c (HbA1c), body weight, and body mass index (BMI) compared with those who performed moderate-intensity continuous training (MICT), though no significant changes in insulin resistance or blood lipid levels were found. Karstoft et al. [16] compared the efficacy of HIIT with energy expenditure-matched continuous-walking training in people with T2D and observed greater improvements in VO_2peak_, body weight, fat mass, and glycemic control with the former. Mitranun et al. [17] also found that HIIT improved HbA1c, maximal aerobic capacity, and other cardiovascular risk factors in T2D patients, even if the total exercise time was reduced to half of that recommended. Similar to the current study, a recent meta-analysis by Jelleyman et al. demonstrated that HIIT is more effective than MICT for improving insulin sensitivity and cardiorespiratory fitness in healthy individuals [18]. However, this study did not determine the suitability of HIIT in individuals with T2D. Indeed, although a few RCTs have demonstrated the efficiency of HIIT in the prevention and treatment of T2D patients, no consensus has yet been reached that HIIT is a superior training protocol for the improvement of glycemic control, body composition, and cardiorespiratory fitness compared with moderate-intensity continuous aerobic training among patients with T2D. Therefore, we performed a meta-analysis to determine the impact of HIIT on body composition, glycemic control, and cardiorespiratory fitness, and to compare it to that of MICT and that of no intervention in randomized-controlled trials in T2D patients, which we hope can provide clinical evidence to enable patients to achieve optimal outcomes.

## Patients and methods

### Search strategy

The databases which we searched included PubMed, the Web of Science, EBSCO, Embase, and the Cochrane Library. All of the databases were searched from their date of inception until April 2018. We included only studies written in English. We used combined key phrases and Medical Subject Heading (MeSH) terms as follows: “type 2 diabetes mellitus,” “diabetes mellitus, type II,” “type 2 diabetes,” “T2D,” “T2DM,” “high-intensity interval training,” “high-intensity aerobic interval exercise,” “high-intensity interval training,” “aerobic interval training,” “high-intensity intermittent exercise,” “HIT,” and “HIIT.” Supporting information appendix in **S1** gives a detailed description of the search strategy. In addition, the reference lists of included studies and reviews were also examined for additional potentially eligible studies.

### Inclusion and exclusion criteria

#### Type of study

This review included studies with randomized-controlled trials. We excluded matched controlled trial designs, uncontrolled trials, observational studies, and animal studies.

#### Type of participant

The study participants were clinically diagnosed with type 2 diabetes. Patients with type 1 diabetes and gestational diabetes were excluded. There was no limitation on the age, gender, or ethnicity of the study participants.

#### Intervention variables and outcome measures

The studies included here were required to report at least one outcome measure, measured at baseline and post-intervention, and compared to either a moderate-intensity exercise intervention or control group. The HIIT program had to be prescribed at least two times per week for 4 weeks, with moderate-intensity continuous training or another treatment (e.g., drug therapy) as the control group.

#### Primary outcomes

Outcome measures included glycemic control (e.g., HbA1c, fasting glucose, and fasting insulin); body composition [e.g., body weight, BMI, body fat (%), and waist circumference]; cardiorespiratory fitness (e.g., VO_2peak_). The criteria which we used complied with the PICO concept (patient/problem/population; intervention; comparison/control/comparator; outcome). For articles reported in more than two publications, only one full copy was used for meta-analysis. Abstracts presented at academic conferences, case reports, observational studies, examples of animal research, and studies of which the full text could not be obtained were excluded.

### Evaluation of bias and quality assessment

The risk of bias and methodological quality of the included trials were assessed independently by two reviewers (Liu and Li), who used the Cochrane Collaboration’s tool [19] to check for concealed allocation, allocation concealment, blinding, incomplete outcome data, selective reporting, and other biases. Each reviewer was required to award one of three grades (either unclear, low risk, or high risk) to each item. The Grading of Recommendations Assessment, Development, and Evaluation (GRADE) system [20] was used to assess the quality of the evidence from very low to high based on risk of bias, inconsistency, indirectness, imprecision, and publication bias. A third reviewer was consulted if any disagreement occurred.

### Data extraction

The two investigators assessed each article’s title or abstract for eligibility. When a disagreement happened, a third investigator participated in a discussion to reach a final consensus. For studies that met the inclusion criteria, full papers were obtained for further analysis. The two authors independently extracted data from the published works using standard data extraction forms. If there were any inconsistencies in the process of data extraction, the two authors would check the original text and reach an agreement through discussion or through verification by a third author. Information on trial design, characteristics of the patients, HIIT protocol, and relevant results was noted according to a redesigned form. We recorded the name of the first author and the year of publication; the number of patients/participants and their ages, gender, and BMIs; the duration of diagnosis; and the experimental and control interventions (e.g., exercise intensity and duration, interval intensity and duration, session time, and duration in weeks). When data were insufficient or inapplicable, we attempted to contact the authors by e-mail or used an equation to reveal all available data.

### Data analysis

The Review Manager software (RevMan 5.3; Cochrane, London, UK) was used to conduct the meta-analysis. The statistical heterogeneity of the treatment effect among the included studies was assessed using the chi-squared test and *I*^2^ test. A threshold of *P* < 0.10 was considered to be statistically significant and an *I*^2^ value > 50% was indicative of high heterogeneity. We used the weighted mean difference (MD) or standardized MD (SMD) with 95% confidence intervals (CIs) for summary statistics and derived such for the comparison of HIIT with MICT or other treatment methods. MD was used when all studies reported the same outcome using the same scale, while SMD was used when studies reported different units or scales for the outcome. If heterogeneity did not exist between studies, we incorporated a fixed-effects model approach to combined outcome measures. A random-effects model was used when there was a large degree of heterogeneity between studies. To account for within-group intervention effect sizes, we used fixed-effects modeling to estimate the change from baseline. Potential heterogeneity sources were identified by sensitivity analyses conducted by omitting one study successively and comparing the influence of each study on the overall pooled estimate if *I*^2^ > 50%.

Data were analyzed using the change from baseline for both groups. If the study did not contain change data, we used the following two equations for conversion:

1 $$M=|{M_1} - {M_2}|,$$

where *M* is the effect mean, *M*_1_ is the mean of the baseline, and *M*_2_ is the end value mean;

2 $${S^2}=S_{1}^{2}+S_{2}^{2} - 2 \times R \times {S_1} \times {S_2},$$

where *S* is the standard deviation of the effect, *S*_1_ is the standard deviation of the baseline value, *S*_2_ is the final standard deviation, and *R* is constant (0.4 or 0.5).

## Results

### Search results

**Fig. 1:**
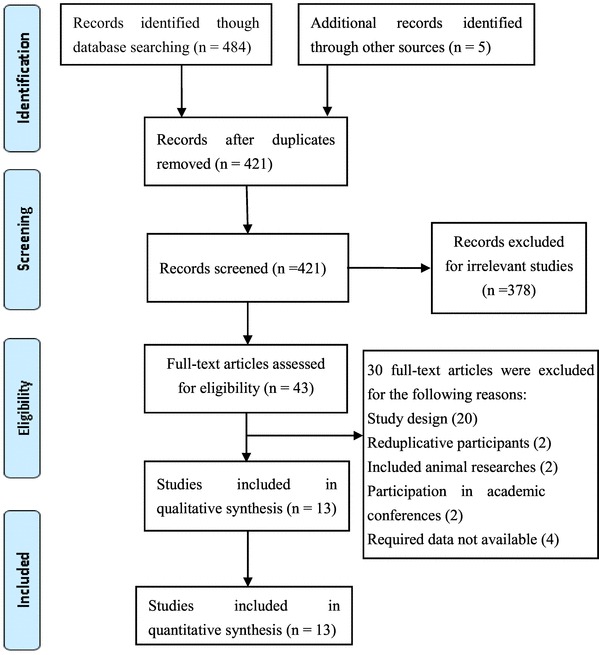
Flowchart of the study selection process

The initial database searches returned a total of 484 articles (PubMed, *n* = 84; EMBASE, *n* = 30; The Cochrane Library, *n* = 63; EBSCO, *n* = 30; the Web of Science, *n* = 277) that were each screened and evaluated for eligibility based on their respective titles only. Following removal of duplicates, 421 articles underwent further identification and screening. In total, 378 non-relevant articles were excluded after screening the titles and abstracts. Of the remaining articles, 43 were selected to be read in full. At this point, 30 additional articles were excluded for varying reasons (e.g., the study was not randomized, there were reduplicative participants, the study was observational in nature, the research was performed on animals, the study was presented at an academic conference, and/or the study had no required data), rendering a final sample of 13 papers. Figure 1 describes the study selection flow.

### Characteristics of included trials

A total of 345 participants were included in the analysis, of which 163 (47.2%) participants underwent a HIIT intervention. The characteristics of the study participants, the HIIT training protocols used, and the main results from the included studies are described in Table 1. The countries or regions of publication were mainly the United Kingdom (*n* = 2), Norway (*n* = 2), the Republic of Korea (*n* = 1), Chile (*n* = 1), Denmark (*n* = 2), France (*n* = 1), Thailand (*n* = 1), Australia (*n* = 1), Italy (*n* = 1), and Canada (*n* = 1). The main HIIT intervention ranged in duration from 11 to 16 weeks (16 weeks in 4 studies, 12 weeks in 8 studies, and 11 weeks in 1 study) and occurred two-to-five times weekly (median: three times). Total training duration per session ranged from 30 s to 4 min, and interval duration ranged from 30 s to 3 min.

**Table 1:** Characteristics of the included trials

Article, year	Country	Main characteristics of the subjects	HIIT		MICT or CON		No. of patient dropouts
			Exercise intensity and interval	Frequency and duration	Exercise intensity	Frequency and duration	
Alvarez 2016 [21]	Chile	HIIT: mean age was 45.6 ± 3.1 years, mean duration of diagnosis was 3.14 ± 1.1 years, mean BMI was 30.8 ± 1.0 kg/m^2^, *n* = 13 CON: mean age was 43.1 ± 1.5 years, mean duration of diagnosis was 3.6 ± 1.1 years, mean BMI was 30.4 ± 0.4 kg/m^2^, *n* = 10	Train: running at 90–100% HR_max_ intensity for 30–120 s; Interval: low-intensity walking for 30–120 s	2.2–37.5 min/time, three times per week for 16 weeks	CON: non-exercise		5
Hollekin 2014 [22]	Norway	Total of 47 patients 55.9 ± 6.0 years; 36% female; mean duration of diagnosis was 3.6 ± 2.5 years HIIT: mean BMI was 30.2 ± 2.8 kg/m^2^, 5% in mild stage, 90% in moderate stage, 5% in severe stage, n = 24 MICT: mean BMI was 29.7 ± 3.7 kg/m^2^, 29.4% in mild stage, 70.6% in moderate stage, *n* = 23	Protocol was 4 × 4 min exercise at 90–95% HR_max_ with 3 min of low-intensity exercise at 70% HR_max_	40 min /bout; three times per week for 12 weeks	MICT: Moderate-intensity aerobics training	210 min per week for 12 weeks	10
Karstoft 2013 [16]	Denmark	HIIT: mean age was 57.5 ± 2.4 years, 41.7% female, mean BMI was 29.0 ± 1.3 kg/m^2^, mean duration of diagnosis was 3.5 ± 0.7 years, *n* = 12 MICT: mean age was 60.8 ± 2.3 years, 33.3% female, mean BMI was 29.9 ± 1.6 kg/m^2^, mean duration of diagnosis was 6.2 ± 1.5 years, *n* = 12 CON: mean age was 60.8 ± 2.3 years, 37.5% females, mean BMI was 29.7 ± 1.9 kg/m^2^, mean duration of diagnosis was 4.5 ± 1.5 years, *n* = 8	Alternating 3 min intervals of fast (≥ 70% of VO_2peak_) and slow (40% of VO_2peak_) walking	Five times per week, 60 min/time for 4 months	MICT: walking at ≥ 55% VO_2peak_ 60 min/session CON: non-exercise	Five times per week, for 4 months	0
Lee 2015 [23]	Korea	Mean age was 15.3 ± 2.2 years, mean BMI was 24.0 ± 3.8 kg/m^2^, mean duration of diagnosis was 4.0 ± 2.2 years, *n* = 20	Exercise at ≥ 80% HRR, train program including 30-s sprint and 30- s recovery	400 Kcal/session, three sessions per week for 12 weeks	MICT: Exercise at ≤ 40% HRR, 200 kcal/session	Six sessions per week for 12 weeks	0
Maillard 2016 [24]	France	Included 16 postmenopausal women with T2D, mean age was 69 ± 1 years, mean BMI was 31 ± 1 kg/m^2^ HIIT: mean age was 68.2 ± 1.9 years, mean BMI was 32.6 ± 1.7 kg/m^2^, *N* = 8 MICT: mean age was 70.1 ± 2.4 years, mean BMI was 29.7 ± 1.2 kg/m^2^, *n* = 8	Repeated cycles of sprinting for 8 s (at around 80% HR_max_) followed by pedaling slowly (20–30 rpm) for 12 s (maximum of 60 cycles per 20-min session)	Two times per week for 16 weeks	MICT: Exercise at 55–60% HRR for 40 min	Two times/week, 16 weeks	1
Mitranun 2014 [17]	Thailand	Total of 43 adults with T2D (64.4% females), HIIT: mean age was 61.2 ± 2.8 years, mean BMI was 29.6 ± 0.5 kg/m^2^, mean duration of diagnosis was 19.5 ± 1.5 years; *n* = 14. MICT: mean age was 61.7 ± 2.7 years, mean BMI was 29.4 ± 0.7 kg/m^2^, mean duration of diagnosis was 20.5 ± 1.5 years; *n* = 14. CON: mean age was 60.9 ± 2.4 years, mean BMI was 29.7 ± 0.4 kg/m^2^, mean duration of diagnosis was 21.1 ± 2.32 years; *n* = 15	Protocol was 1-min high-intensity exercise at 50–85% VO_2peak_ with 4-min low-intensity at 50–60% VO_2peak_ interval	20 min /session, three sessions per week for 12 weeks	MICT: exercise intensity at 50–60% VO_2peak_ for 25–30 min	Three times/week, 12 weeks CON: non-exercise	2
Ramos 2016 [25]	Australia	HIIT: mean weight was 99 ± 18 kg, *n* = 9 MICT: mean weight was 98 ± 17 kg, *n* = 6	Protocol was 4*4 min bouts at 85–95% HR_max_, interval with 3 min of active recovery at 50–70% HR_max_	38 min per session, 3 times per week for 16 weeks	MICT: 30 min at 60–70% HR_peak_	5 times/week, 16 weeks	0
Støa 2017 [15]	Norway	HIIT: mean age was 59 ± 11 years, mean BMI was 32.0 ± 4.7 kg/m^2^, mean duration of diagnosis was 9 ± 7 years, *n* = 19 MICT: mean age was 59 ± 10 years, mean BMI was 31.1 ± 4.5 kg/m^2^, mean duration of diagnosis was 6 ± 5 years, n = 19	Protocol was 4*4 min of walking or uphill at 85–95% of HR_max_	Three times per week for 12 weeks	MICT: 60-min walking at 70–75% of HR_peak_	Three times per week for 12 weeks	5
Terada 2013 [26]	Canada	HIIT: mean age was 62 ± 3 years, mean BMI was 28.4 ± 4.1 kg/m^2^, 50% female, mean duration of diagnosis was 6 ± 4 years, *n* = 8 MICT: mean age was 63 ± 5 years, mean BMI was 33.1 ± 4.5 kg/m^2^, 42.9% female, mean duration of diagnosis was 8 ± 74 years, *n* = 7	HIIT protocol involved alternating 1-min intervals 100% VO_2peak_ with 3-min recovery intervals at 20% VO_2peak_	30–60 min/day, 5 days per week for 12 weeks	MICT: continuous exercise at 40% VO_2peak_, 30–60 min/day	5 days per week for 12 weeks	0
Backx 2011 [27]	UK	Involved 15 males and 4 females; total median age was 59.6 (44.0–69.0) years HIIT: median BMI was 30.0 (25.3–40.1) kg/m^2^, *n* = 10 MICT: median BMI was 32.3 (26.4–40.5) kg/m^2^, *n* = 9	Protocol was 1–2 min at 40–50% HRR and 1, 2, or 3 min at 80–90% HRR	60 min/day, 3 days per week for 12 weeks	MICT: Exercise at moderate-to-high-intensity for 30 min	Five times/week, 12 weeks	2
Cassidy 2016 [28]	UK	HIIT: mean age was 61 ± 9 years, mean BMI was 31 ± 5 kg/m^2^, mean duration of diagnosis was 5 ± 3 years, *n* = 12 MICT: mean age was 59 ± 9 years, mean BMI was 32 ± 6 kg/m^2^, mean duration of diagnosis was 4 ± 2 years, *n* = 11	Training: Pedal cadence > 80 rev/min, ranching a RPE 16–17 (very hard); interval: 3-min recovery cycle	Three sessions per week for 12 weeks	Non-exercise		5
Bellia 2017 [29]	Italy	HIIT: mean age was 58.8 ± 7.9, mean BMI was 27.7 ± 2.8 kg/m^2^, mean duration of diagnosis was 5.9 ± 4.4 years, *n* = 11 CON: mean age was 56.3 ± 6.4, mean BMI was 29.9 ± 3.4 kg/m^2^, mean duration of diagnosis was 3.4 ± 3.7 years, *n* = 11	Protocol involved a 4-min walk at 75–80% HR_max_ to be repeated two-to-four times, interval with 3-min active recovery at 45–50% HR_max_	Two–three times per week for 12 weeks	MICT: protocol was 10,000 steps per day or 70,000 steps per week for 12 weeks		7
Winding 2018 [30]	Denmark	HIIT: mean age was 54 ± 6, mean BMI was 28.1 ± 3.5 kg/m^2^, mean duration of diagnosis was 8 ± 4 years, *n* = 13 MICT: mean age was 58 ± 8 years, mean BMI was 27.4 ± 3.1 kg/m^2^, mean duration of diagnosis was 6 ± 4 years, *n* = 12 CON: mean age was 57 ± 7, mean BMI was 28 ± 3.5 kg/m^2^, mean duration of diagnosis was 7 ± 5 years, *n* = 7	Training: initiated with a 20 min of cycling consisting of cycles of 1 min at 95% W_peak_ and 1 min of active recovery (20% W_peak_) was performed	3 days per week for 11 weeks	MICT: 40 min of cycling at 50% of Wpeak	3 days/week, 11 weeks	3

*RCT* randomized-controlled trial, *MCT* Matched controlled trial designs, *HR*_*max*_ maximal heart rate, *HIIT* high-intensity interval training, *MICT* moderate-intensity continuous training, *CON* control, *HRR* heart rate reserve, *RPE* rating of perceived exertion

### Risk of bias among the selected articles

The 13 studies were assessed for risk of bias; the evaluation results are shown in Table 2. Among the included studies, the method of randomization was only clearly stated in four studies [21, 25, 26, 28], while three reported allocation concealment [25, 26, 28], five blinded participants or personnel [15, 16, 21, 23, 28], and three did not employ assessor blinding [22, 24, 27]. Only one study did not clearly state complete outcomes data and employed selective reporting [22]; no other bias in all studies. The evaluation of the overall quality of evidence and results is shown in Table 4, and the level of evidence for RCTs is downgraded due to inconsistency and imprecision in most of the studies.

**Table 2:** Risk-of-bias assessment for the included studies

Study	Random sequence generation	Allocation concealment	Blinding		Incomplete outcome data	Selective reporting	Other bias
			Participants or personnel	Outcome assessment			
Alvarez [21]	Low	Unclear	Low	Low	Low	Low	Low
Hollekin [22]	Unclear	Unclear	Unclear	Unclear	Unclear	Unclear	Low
Karstoft [16]	Unclear	Unclear	Low	Low	Low	Low	Low
Lee [23]	Unclear	Unclear	Unclear	Low	Low	Low	Low
Maillard [24]	Unclear	Unclear	Unclear	Unclear	Low	Low	Low
Mitranun [17]	Unclear	Unclear	Unclear	Low	Low	Low	Low
Ramos [25]	Low	Low	Low	Low	Low	Low	Low
Støa [15]	High	High	Low	Low	Low	Low	Low
Terada [26]	Low	Low	High	Low	Low	Low	Low
Backx [27]	High	Unclear	Unclear	Unclear	Low	Low	Low
Cassidy [28]	Low	Low	Low	Low	Low	Low	Low
Bellia [29]	Unclear	Unclear	Unclear	Low	Low	Low	Low
Winding [30]	Unclear	Unclear	Unclear	Low	Low	Low	Low

### Effects of HIIT on body composition

The included studies assessed body weight (11/13; 84.6%); BMI (11/13; 84.6%); body fat (6/13; 46.2%); waist circumference (7/13; 53.8%) as outcomes. Of these, 8/11 (72.7% of body weight studies); 8/11 (72.7% of BMI studies); 5/6 (83.3% of body fat studies); 6/7 (85.7% of waist circumference studies) compared HIIT to MICT. The meta-analyses showed (Table 3) a significant reduction in body weight of 1.22 kg [95% CI − 2.23 to − 0.18, *P* = 0.02] for patients in the HIIT group as compared with those in the MICT group. Furthermore, in comparison with baseline, there was a reduction in BMI of 0.85 kg/m^2^ (95% CI − 1.57 to − 0.12, *P* = 0.02) (Table 3), and, as compared with the MICT group, the reduction was 0.40 kg/m^2^ (95% CI − 0.78 to − 0.02, *P* = 0.04) (Table 3). In addition, as compared with baseline, there was a reduction in body fat of 1.86% (95% CI − 3.68 to − 0.04, *P* = 0.02) (Table 3), but the reduction was not statistically significant as compared with that in the MICT group. In addition, there was no significant difference in the waist circumference reduction following HIIT versus MICT or at baseline (Table 3).

**Table 3:** Effect of HIIT on body composition, glycemic control, lipid control, and cardiorespiratory fitness in patients with T2D

Body composition		Within groups	Compared to CON	Compared to MICT
Body weight	*N*	11	6	8
	ES (95% CI)	MD: − 1.65 [− 4.76, 1.46]	MD: − 0.78 [− 2.36, 0.80]	MD: − 1.22 [− 2.23, − 0.18]
	*I*^2^ (%)	0	0	0
BMI	*N*	11	4	8
	ES (95% CI)	MD: − 0.85 [− 1.57, − 0.12]	MD: − 0.80 [− 1.86, 0.27]	MD: − 0.40 [− 0.78, − 0.02]
	*I*^2^ (%)	0	0	0
Body fat (%)	*N*	6	ND	5
	ES (95% CI)	MD: − 1.86 [− 3.68, − 0.04]		MD: − 0.50 [− 1.18, 0.19]
	*I*^2^ (%)	0		0
Waist circumference	*N*	7	ND	6
	ES (95% CI)	MD: − 2.23 [− 5.00, 0.55]		MD: − 0.15 [− 1.21, 0.91]
	*I*^2^ (%)	0		0
Glycemic control				
HbA1c (%)	*N*	10	3	9
	ES (95% CI)	MD: − 0.29 [− 0.55, − 0.04]	MD: − 0.39 [− 0.81, 0.02]	MD: − 0.37 [− 0.55, − 0.19]
	*I*^2^ (%)	0	0	0
Fasting glucose	*N*	9	5	8
	ES (95% CI)	MD: − 0.41 [− 0.91, 0.09]	SMD: − 0.31 [− 0.69, 0.06]	MD: 0.10 [− 0.84, 0.65]
	*I*^2^ (%)	0	0	0
Fasting insulin	*N*	6	5	4
	ES (95% CI)	SMD: − 0.46 [− 0.81, − 0.11]	SMD: − 0.46 [− 0.91, 0.02]	SMD: − 0.19 [− 0.58, 0.20]
	*I*^2^ (%)	41	26	0
HOMA-IR	*N*	7	4	6
	ES (95% CI)	MD: − 0.43 [− 1.18, 0.32]	MD: − 0.18 [− 0.79, 0.42]	MD: 0.13 [− 0.10, 0.36]
	*I*^2^ (%)	73	0	0
Lipid control				
Total cholesterol	*N*	8	6	7
	ES (95% CI)	SMD: − 0.13 [− 0.42, 0.15]	SMD: 0.02 [− 0.32, 037]	MD: − 0.18 [− 0.44, 0.07]
	*I*^2^ (%)	0	9	0
HDL cholesterol	*N*	11	5	9
	ES (95% CI)	SMD: 0.20 [− 0.07, 0.48]	SMD: 0.60 [− 0.26, 1.45]	MD: − 0.04 [− 0.10, 0.02]
	*I*^2^ (%)	39	83	0
LDL cholesterol	N	7	5	6
	ES (95% CI)	SMD: − 0.15 [− 0.44, 0.13]	MD: − 0.60 [− 1.74, 0.54]	MD: − 0.25 [− 0.46, − 0.04]
	*I*^2^ (%)	0	52	0
Cardiorespiratory fitness				
VO_2peak_ (ml/kg/min)	*N*	7	2	7
	ES (95% CI)	MD: 4.75 [2.94, 6.56]	MD: 4.12 [2.66, 5.57]	MD: 3.37 [1.88, 4.87]
	*I*^2^ (%)	0	0	48
VO_2peak_ (L/min)	*N*	5	2	6
	ES (95% CI)	MD: 0.35 [0.17, 0.53]	MD: 0.24 [0.10, 0.37]	MD: 0.37 [0.28, 0.45]
	*I*^2^ (%)	0	0	36

*ES* effect sizes, *CI* confidence interval, *MD* mean difference, *SMD* standardized mean difference, *ND* not enough data

### Effects of HIIT on glycemic control

**Fig. 2:**
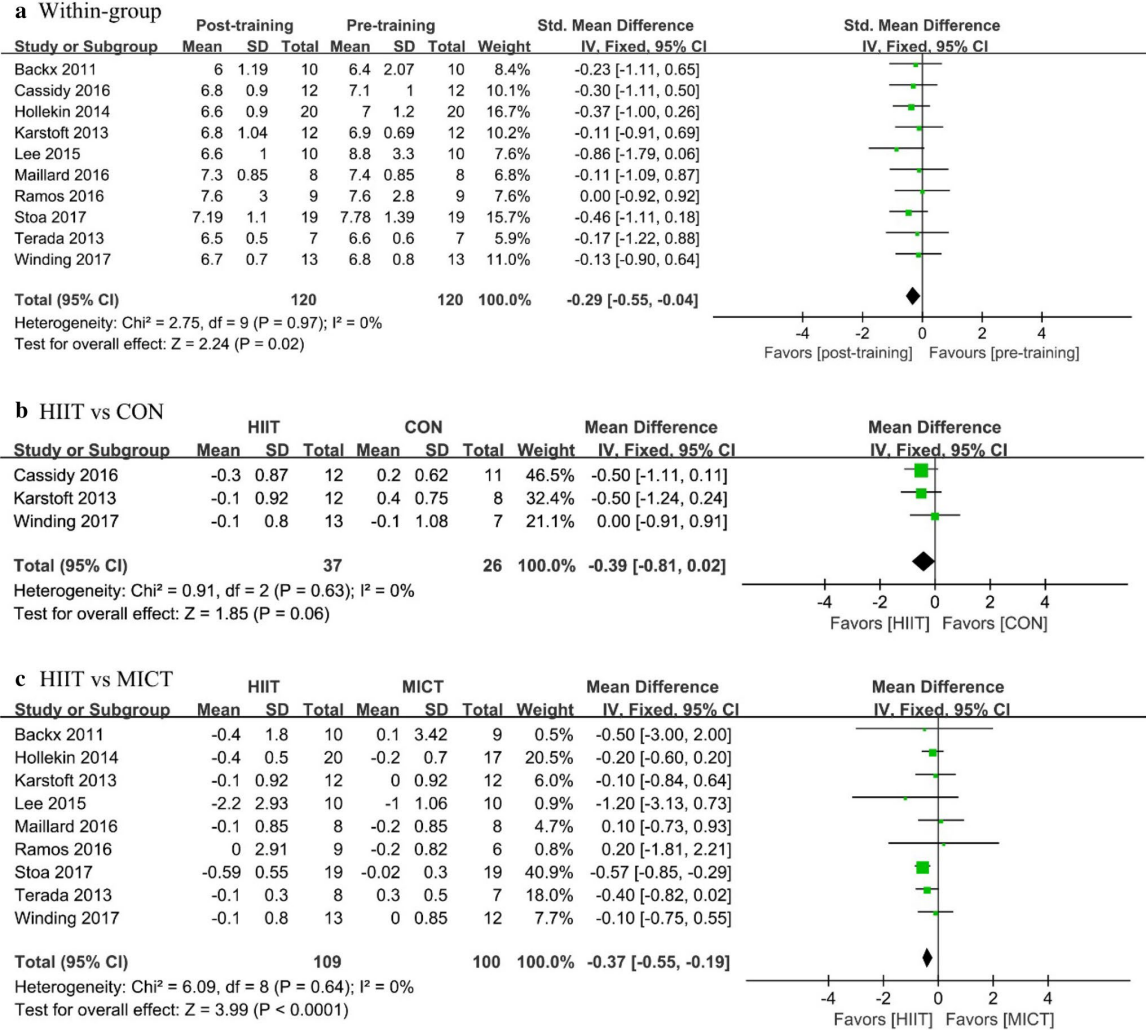
Forest plot for change in of HbA1c (%), **a** before and after (within-group) high-intensity interval training (HIIT), **b** between HIIT and control (CON) intervention, and **c** between HIIT and moderate-intensity training (MICT)

Ten studies with 220 patients assessed HbA1c. Of these, nine studies compared changes in HbA1c in HIIT groups to those in MICT groups, while only three studies compared such to changes in CON groups. Relative to baseline, there was a significant reduction in HbA1c (SMD: − 0.29, 95% CI − 0.55 to − 0.04, *P* = 0.02) (Fig. 2a; Table 3). Compared with MICT, the reduction was 0.37% (95% CI − 0.55 to − 0.19, *P* < 0.0001, Fig. 2a). However, in comparison with a control intervention, a non-significant change in HbA1c of -0.39% (95% CI − 0.81 to 0.02, *P* < 0.06, Fig. 2b) was found. As compared with baseline, there was a significant reduction in fasting insulin (SMD: − 0.46, 95% CI − 0.81 to − 0.11, *P* = 0.01, Table 3). However, this reduction was not significantly different as compared with that in the control intervention or MICT groups (Table 3). No significant difference in the fasting glucose or HOMA-IR (homeostatic model assessment of insulin resistance) was found for participants in the HIIT group as compared with those in the MICT group (Table 3). We further used sensitivity analysis in HOMA-IR because of the larger heterogeneity (*I*^2^ = 73%) within the group. The results of sensitivity analysis showed that the heterogeneity (*I*^2^ = 0%) was significantly reduced after exclusion of Lee 2015, but there was no significant change in results.

### Effects of HIIT on lipid control

Seven studies assessed low-density lipoprotein (LDL) cholesterol as an outcome. Of these, five studies compared the change in the HIIT group to that in the control group and six studies compared the change in the HIIT to that in the MICT group. There was also a significant reduction in LDL cholesterol (MD: − 0.25 mmol/L 95% CI − 0.46 to − 0.04, *P* = 0.02) with HIIT versus with the MICT group (Table 3). Unfortunately, there was no significant change in total cholesterol as compared with both the control and MICT groups and a similar result was found with respect to high-density lipoprotein (HDL) cholesterol. LDL cholesterol did not differ significantly between the HIIT group and the control group. Because studies comparing HIIT with control interventions in relation to LDL and HDL cholesterol showed significantly more heterogeneity, we conducted sensitivity analysis that showed that the studies heterogeneity changed significantly (*I*^2^ = 20% in LDL cholesterol, *I*^2^ = 0 in HDL cholesterol) after the removal of Alvarez 2016, but there were no significant changes in the results.

### Effects of HIIT on cardiorespiratory fitness

**Fig. 3:**
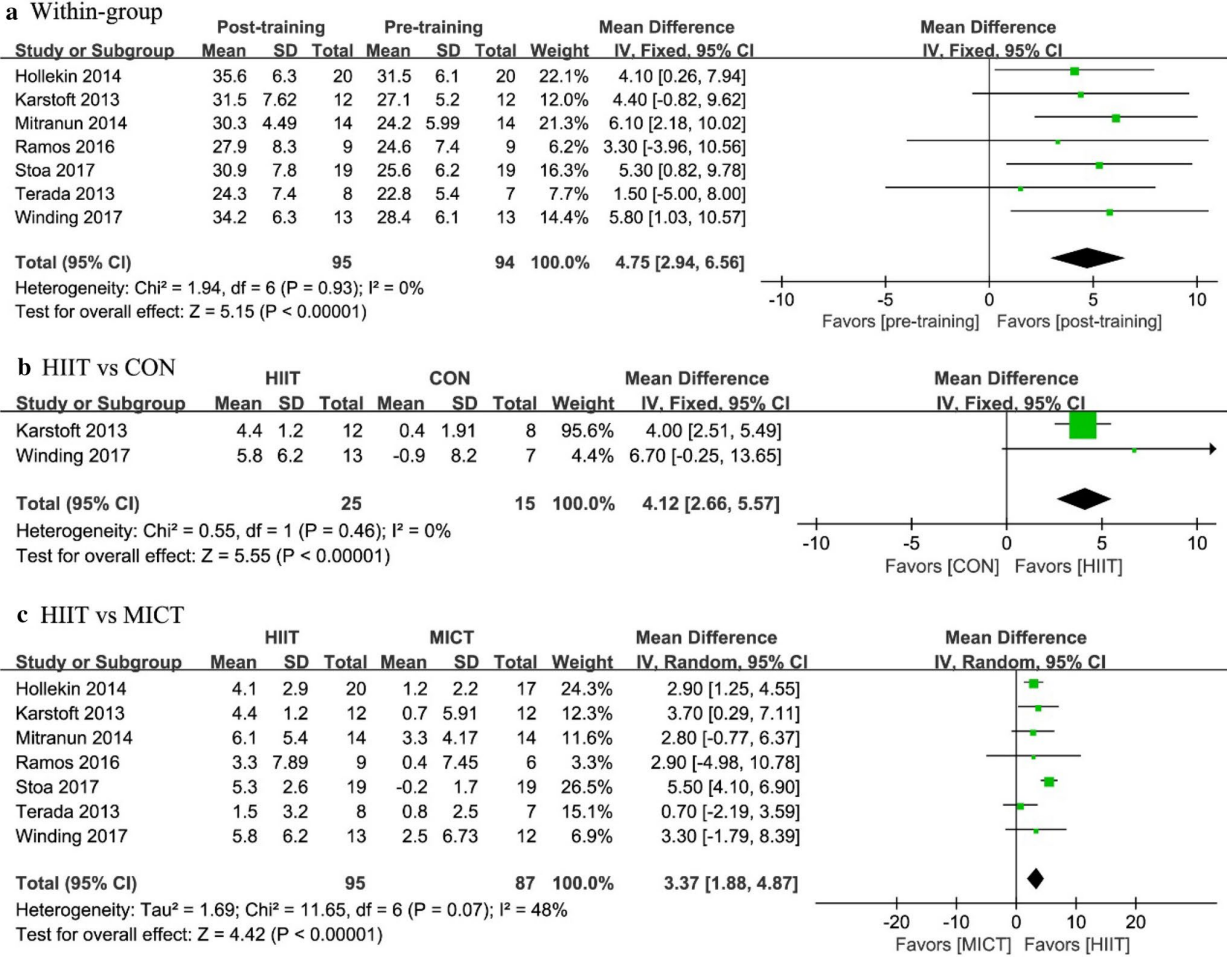
Forest plot for change in VO_2peak_ (ml/kg/min), **a** before and after (within-group) high-intensity interval training (HIIT), **b** between HIIT and control (CON) intervention, and **c** between HIIT and moderate-intensity training (MICT)

**Fig. 4:**
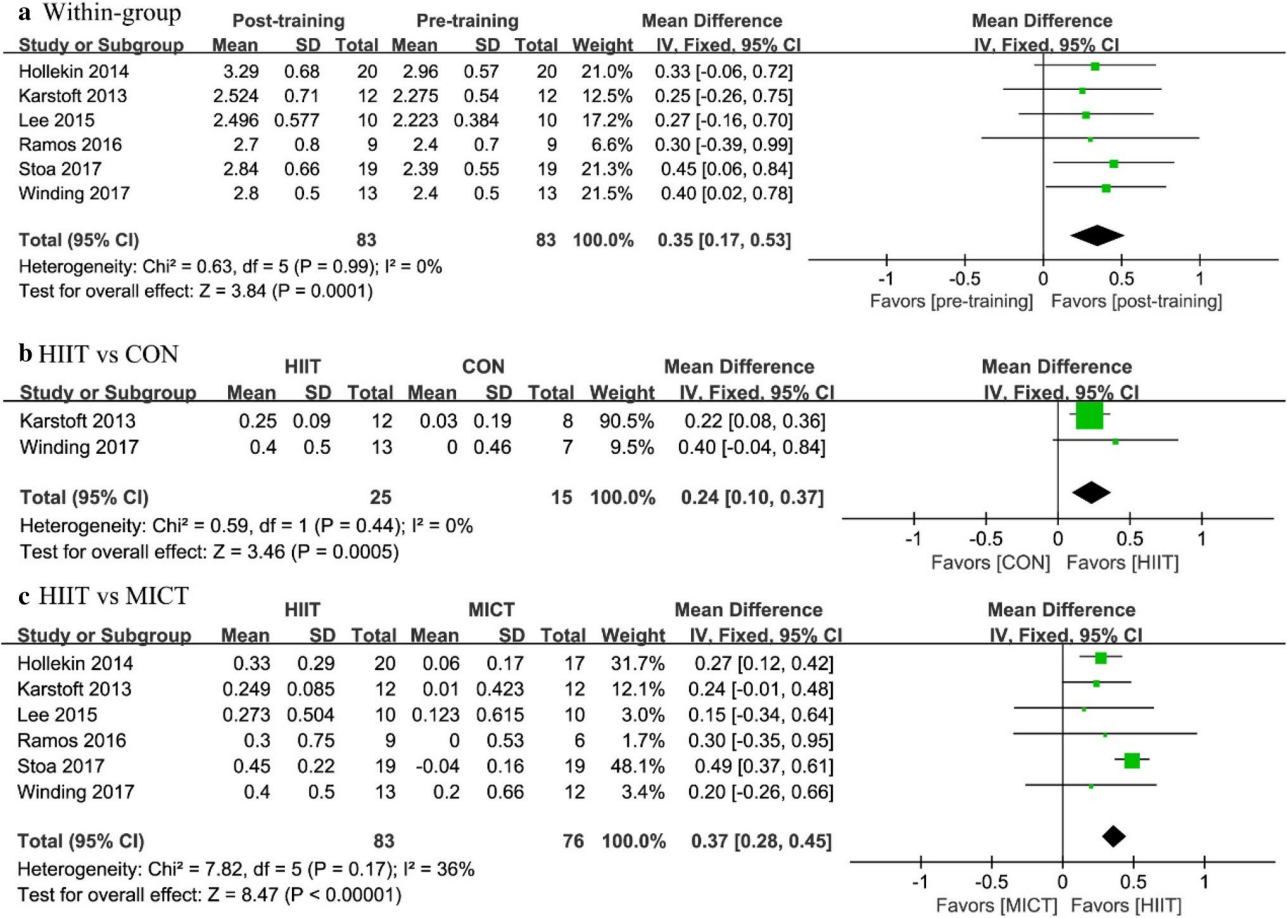
Forest plot for change in VO_2peak_ (L/min), **a** before and after (within-group) high-intensity interval training (HIIT), **b** between HIIT and control (CON) intervention, and **c** between HIIT and moderate-intensity training (MICT)

Cardiorespiratory fitness as measured using absolute VO_2peak_ (L/min) and relative VO_2peak_ (ml/kg/min) was analyzed using data from seven studies representing a total of 219 patients. As compared with baseline, there was a 4.75 ml/kg/min (95% CI 2.94 to 6.56, *P* < 0.0001) (Fig. 3a; Table 2) or 0.35 L/min (95% CI 0.17 to 0.53, *P* = 0.0001) increase in VO_2peak_ with HIIT (Fig. 4a; Table 3). In addition, there was a 4.12 ml/kg/min (95% CI 2.66 to 5.57, *P* < 0.0001) (Fig. 3b) or 0.24 L/min (95% CI 0.10 to 0.37, *P* = 0.0005) (Fig. 4b) increase in VO_2peak_ with HIIT over control interventions. The random-effects model showed (Fig. 4c) a significant improvement in absolute VO_2peak_ of 0.37 L/min (95% CI 0.28 to 0.45, *P* < 0.0001) for patients in HIIT group versus those in the MICT group and there was a similar increase seen with respect to relative VO_2peak_ (MD: 3.37 ml/kg/min, 95% CI 1.88 to 4.87, *P* < 0.0001) (Fig. 3c). However, there existed moderate heterogeneity in this analysis (*I*^2^ = 48%) and the results should be interpreted with caution (Table 4).

**Table 4:** Summary of GRADE’s approach to rating quality of evidence

Outcomes	Quality assessment							
	Comparison	Participants (studies) follow up	Risk of bias	Inconsistency	Indirectness	Imprecision	Publication bias	Overall quality of evidence
Body weight	MICT	185 (eight studies)	None	None	Serious	Serious	Undetected	⊕⊕⊝⊝ Low due to indirectness and imprecision
	CON	136 (six studies)	None	None	Serious	Serious	Undetected	⊕⊕⊝⊝ Low due to indirectness and imprecision
BMI	MICT	207 (eight studies)	None	None	Serious	Serious	Undetected	⊕⊕⊝⊝ Low due to indirectness and imprecision
	CON	72 (three studies)	None	None	Serious	Serious	Undetected	⊕⊕⊝⊝ Low due to indirectness and imprecision
Body fat (%)	MICT	138 (five studies)	Serious	Very serious	None	Serious	Undetected	⊕⊝⊝⊝ Very low due to risk of bias, inconsistency and imprecision
	CON	ND	ND	ND	ND	ND	ND	ND
Waist circumference	MICT	140 (six studies)	None	Serious	None	Serious	Undetected	⊕⊕⊝⊝ Low due to inconsistency and imprecision
	CON	ND	ND	ND	ND	ND	ND	ND
HbA1c (%)	MICT	209 (nine studies)	None	Serious	None	Serious	Undetected	⊕⊕⊝⊝ Low due to inconsistency, imprecision
	CON	63 (three studies)	None	Serious	None	Serious	Undetected	⊕⊕⊝⊝ Low due to inconsistency and imprecision
Fasting glucose	MICT	162 (eight studies)	None	Serious	None	Serious	Undetected	⊕⊕⊝⊝ Low due to inconsistency and imprecision
	CON	114 (five studies)	None	Serious	None	Serious	Undetected	⊕⊕⊝⊝ Low due to inconsistency and imprecision
Fasting insulin	MICT	103 (five studies)	None	Serious	None	Serious	Undetected	⊕⊕⊝⊝ Low due to inconsistency and imprecision
	CON	85 (four studies)	None	Serious	None	Serious	Undetected	⊕⊕⊝⊝ Low due to inconsistency and imprecision
HOMA-IR	MICT	182 (seven studies)	None	Very serious	None	Serious	Undetected	⊕⊝⊝⊝ Very low due to inconsistency and imprecision
	CON	99 (four studies)	None	Very serious	None	Serious	Undetected	⊕⊝⊝⊝ Very low due to inconsistency and imprecision
Total cholesterol	MICT	165 (seven studies)	None	Serious	None	Serious	Undetected	⊕⊕⊝⊝ Low due to inconsistency and imprecision
	CON	137 (six studies)	None	Serious	None	Serious	Undetected	⊕⊕⊝⊝ Low due to inconsistency and imprecision
HDL cholesterol	MICT	204 (nine studies)	None	Serious	None	Serious	Reporting bias strongly suspected	⊕⊝⊝⊝ Very low due to inconsistency, imprecision and publication bias
	CON	114 (five studies)	None	Serious	None	Serious	Reporting bias strongly suspected	⊕⊝⊝⊝ Very low due to inconsistency, imprecision and publication bias
LDL	MICT	150 (six studies)	None	Serious	None	Serious	Reporting bias strongly suspected	⊕⊝⊝⊝ Very low due to inconsistency, imprecision and publication bias
	CON	114 (five studies)	None	Very serious	None	Serious	None	⊕⊝⊝⊝ Very low due to inconsistency and imprecision
VO2peak (L/min)	MICT	159 (six studies)	None	Serious	None	Serious	Undetected	⊕⊕⊕⊝ Moderate due to inconsistency, imprecision and large effect
	CON	40 (two studies)	None	Serious	None	Serious	Undetected	⊕⊕⊝⊝ Low due to inconsistency and imprecision
VO2peak (ml/kg/min)	MICT	182 (seven studies)	Serious	Serious	None	Serious	Undetected	⊕⊕⊕⊝ Moderate due to inconsistency, imprecision and large effect
	CON	40 (two studies)	None	Serious	None	Serious	Undetected	⊕⊕⊝⊝ Low due to inconsistency and imprecision

## Discussion

The purpose of this study was to evaluate the effectiveness of HIIT on body composition, glycemic control, and cardiorespiratory fitness in patients with T2D; to observe the difference in such compared with MICT or non-exercise; and to provide information on an ideal time-efficient physical activity program. The principal finding of the current meta-analysis was that HIIT was more efficient than MICT in increasing VO_2peak_ in T2D patients; they also found that reduction of BMI, body weight, and HbA1c (%) was less conclusive because of low quality of the evidence.

Excess weight and obesity are important risk factors for the occurrence of T2D and contribute to the development of insulin resistance in obese individuals [31, 32]. Even with a body weight that falls within the normal range, individuals with an abnormal BMI and waist circumference can also present with an increased risk of abnormal glucose metabolism [33]. Our work showed that HIIT improved body composition, reducing BMI significantly by 0.85 kg/m^2^ and reducing body fat by 1.86%. Notably, both body weight and BMI were significantly decreased compared with the MICT group, which suggests that HIIT may be more effective for improving body composition (even in the absence of changes in body fat and waist circumference) in individuals with T2D. The underlying mechanism of HIIT-induced body weight loss may be related to the consumption and release of fat from visceral fat stores. Maillard et al. [24] studied and compared the effects of HIIT and MICT on abdominal fat in postmenopausal women with T2D, and observed that only HIIT reduced the subcutaneous and visceral fat mass significantly following 16 weeks of training. Cassidy et al. [28] reported, in their randomized study, that there was a 39% relative reduction in liver fat following HIIT performance and observed that there was a significant correlation with changes in HbA1c and 2-h glucose. Moreover, Karstoft et al. [16] found that patients with T2D had greater oxygen consumption during HIIT training than did those who performed MICT, suggesting that this may be responsible for their greater weight loss. Recent studies have shown that the positive effects of exercise on body composition may be related to the improvement of glycemic control. For example, in a long-term randomized trial, Senechal et al. [34] found that changes in HbA1c were associated with changes in body weight, waist circumference, and trunk fat mass in individuals with T2D. Notably, however, although this review shows that HIIT has favorable effects on body fat reduction in individuals with T2D, the effects of HIIT on blood lipids were limited. Only LDL cholesterol showed significantly lower levels after HIIT than after MICT, while total cholesterol and HDL cholesterol did not. Thus, more studies are required to determine whether HIIT could be a successful training program for lipid control in T2D patients.

HbA1c is not only the most widely used indicator of glucose: it is also an important risk factor of cardiovascular disease in patients with T2D [35, 36]. The previous studies have shown that if HbA1c levels are reduced by 1%, the risk of microvascular complications is reduced by 37% and that of death related to diabetes can be reduced by 21% [35]. A recent meta-analysis has shown that an increase of 100 min in physical activity per week was associated with an average change of − 0.16% of HbA1c in individuals with T2D and pre-diabetes subjects [37]. In our meta-analysis, HbA1c (%) was found to be lower after HIIT than at baseline (SMD: − 0.29, 95% CI − 0.55 to − 0.04). Similar to our findings, a recent meta-analysis of RCTs by Grace et al. identified the positive effects of aerobic exercise in reducing HbA1c levels over with controls [11]. HIIT showed a 0.37% greater reduction of HbA1c than MICT, which means that HIIT may have additional benefits on glycemic control. This is inconsistent with the findings of a meta-analysis conducted by Jelleyman et al. [18], which found that, while HIIT can reduce the levels of HbA1c in patients with diabetes and metabolic syndrome, there is no significant difference in reduction versus with continuous training. Furthermore, in a previous review with a meta-analysis, it was concluded that exercise intensity was a better predictor of weight MD in HbA1c than exercise volume in T2D patients [38]. Unfortunately, we noted no difference in fasting glucose, fasting insulin, or insulin resistance changes in patients following HIIT as compared with the CON and MICT groups, even though the previous studies have shown that the effects of aerobic training on insulin intensity are more closely influenced by high-exercise intensity than by low- or moderate-intensity exercise [39]. The inconsistent results could partly be explained by the difference among methods used to measure insulin sensitivity, as well as the difference in the baseline of glycemic control. Further research would need to include data on the HIIT intervention program (e.g., training intensity, duration of interval time, frequency of training, and total duration) and the characteristics of patients (especially with respect to age, duration of diabetes, and the baseline glycemic control), which all impact trial results.

Both VO_2peak_ and HbA1c are important predictors of cardiovascular events in T2D patients [35], and the previous studies have shown that low cardiorespiratory fitness was associated with an increased risk for impaired glycemic control [40, 41]. Aerobic exercise training represents an effective means to improve VO_2peak_ and HbA1c, and a previous meta-analysis has revealed that aerobic exercise intensity is the primary stimulus for improved VO_2peak_ in people with T2D [11]. Our study further compared the difference between HIIT and MICT in increasing peak VO_2_ and found that the improvement of 3.37 ml/kg/min in relative VO_2peak_ and 0.37 L/min in absolute VO_2peak_ following HIIT is superior to those seen with MICT. Our findings are similarly to those from other recent studies. A meta-analysis focused mainly on cardiac patients by Xie et al. [42] showed that HIIT is more effective than continuous training in improving VO_2peak_ [MD: 1.76 ml/kg/min, 95% CI 1.06 to 2.46 ml/kg/min]. Another systemic analysis analyzing 65 studies by Batacan et al. [43] revealed that HIIT yielded a significant increase in VO_2peak_ by a large amount in normal-weight populations and a medium effect in overweight/obese populations, with an aggregate improvement of 3.8 and 4.43 ml/kg/min, respectively. A more recent meta-analysis including 594 coronary artery disease patients by Gomes-Noto et al. [44] reported that a higher improvement in VO_2peak_ (MD: 1.3 ml/kg/min, 95% CI 0.6 to 1.9 ml/kg/min) was seen with HIIT versus with MICT. The underlying physiological mechanisms of HIIT that improve peak VO_2_ could not be ascertained from the present study, but may involve a combination of central and peripheral adaptations, including an increase in cardiac output, an improvement in vascular/endothelial function, and increased muscle oxidation, which together promote the enhanced availability, extraction, and use of oxygen during exercise [45, 46]. Revdal et al. [47] studied the impact of HIIT on cardiac structure and function in T2D patients, and observed a 12% relative increase in left-ventricular wall mass and increased end-diastolic blood volume, thus demonstrating improvements in systolic function, as indicated by raised stroke volume and left-ventricular ejection fraction. A similar finding was found by Hollekin et al. [22], who observed that both MICT and HIIT groups showed improved diastolic function at rest, but that the HIIT group showed greater improvement than did the MICT group. Moreover, Little et al. [48] found that people with T2D who performed six sessions of low-volume HIIT at an intensity of 90% of the maximal heart rate with 60-s rest over 2 weeks experienced an increase in maximal activity of citrate synthesis and skeletal muscle mitochondrial protein content, suggesting that the increases in skeletal muscle mitochondrial content and function following low-volume HIIT may be contributing factors to improved VO_2peak_.

### Strengths and limitations

Our meta-analysis of randomized trials has several strengths. First, to our knowledge, this is the first existing systematic review to compare the effects of HIIT and MICT or non-exercise on glycemic control (e.g., HbA1c, insulin, and fasting glucose); body composition (e.g., body weight, body fat, BMI, and waist circumference); and cardiorespiratory fitness (e.g., VO_2peak_) among people with T2D. Second, this systematic review involved a large number of literature searches by two reviewers who independently screened studies, assessed their quality, and extracted data to decrease publishing bias and increase credibility.

However, some limitations were still present in our evaluation. First, there are some inconsistencies among the included studies with respect to HIIT protocols and MICT protocols, which may have affected the results obtained with respect to the intervention and control groups. Second, considering the low quality of evidence, these results may have some limitations in guiding clinical applications. Third, an important limitation is that most of the included studies reported the pre- and post-intervention parameters but not the differences between the baselines. Therefore, considering the different baseline values that may be present between the intervention and control groups in some studies, we used equations to calculate the mean difference whenever it was not reported to address the discrepancy of the baseline in each group, and this could have resulted in a bias. Fourth, the results of this meta-analysis are limited by the lack of high-quality studies and the small number of patients in each included study. Only four of the included studies clearly indicated random sequence generation, while three studies reported allocation concealment, and five studies blinded participants in their experimental procedures.

## Conclusions

In conclusion, we here demonstrated that HIIT is an effective strategy for improving cardiorespiratory fitness in patients with T2D, preferable to MICT. Results related to other parameters associated with the prognosis of T2D, such as HbA1c, body weight, and BMI, were not conclusive. This review can still provide some suggestions for the clinical application of HIIT in T2D patients. Future studies should investigate the effects of HIIT in T2D patients through multicenter RCTs with large sample sizes over the long term.
